# Myosin VI and Optineurin Are Required for Polarized EGFR Delivery and Directed Migration

**DOI:** 10.1111/j.1600-0854.2010.01101.x

**Published:** 2010-08-03

**Authors:** Margarita V Chibalina, Alexei Poliakov, John Kendrick-Jones, Folma Buss

**Affiliations:** 1 Cambridge Institute for Medical Research, Department of Clinical Biochemistry, University of Cambridge, Wellcome Trust/MRC BuildingHills Road, Cambridge, CB2 0XY, UK; 2 Division of Developmental Neurobiology, Medical Research Council National Institute for Medical ResearchLondon, NW7 1AA, UK; 3 MRC Laboratory of Molecular BiologyHills Road, Cambridge, CB2 2QH, UK

**Keywords:** cell migration, EGFR, leading edge, myosin VI, optineurin

## Abstract

The polarized trafficking of membrane proteins into the leading edge of the cell is an integral requirement for cell migration. Myosin VI and its interacting protein optineurin have previously been shown to operate in anterograde trafficking pathways, especially for the polarized delivery of cargo to the basolateral domain in epithelial cells. Here we show that in migratory cells ablation of myosin VI or optineurin inhibits the polarized delivery of the epidermal growth factor receptor (EGFR) into the leading edge and leads to profound defects in lamellipodia formation. Depletion of either myosin VI or optineurin, however, does not impair the overall ability of cells to migrate in a random migration assay, but it dramatically reduces directed migration towards a growth factor stimulus. In summary, we identified a specific role for myosin VI and optineurin in directionally persistent cell migration, which involves the polarized delivery of vesicles containing EGFR into the leading edge of the cell.

Migration is a fundamental cellular activity that is crucial for a wide range of cellular events that includes embryonic development, wound healing, inflammation and cancer cell dissemination and metastasis. Cell membrane protrusion at the leading edge and subsequent migration involve a complex set of processes initiated by extracellular signals that trigger regulated actin remodelling at the plasma membrane together with endocytic/exocytic membrane delivery. To initiate forward migration, the cell polarizes along the axis of movement and forms protrusions at its leading edge. These protrusions are stabilized by the assembly of cell-substrate adhesion sites, whereas at the trailing rear end of the cell actomyosin II-dependent contraction releases these adhesions and drives the cell body forward (1). The formation of membrane protrusions at the leading edge is dependent on actin filament polymerization that is regulated by the Arp2/3 complex and the Rho family of small GTPases; e.g. Rac mediates the formation of broad lamellipodia, whereas cdc42 activation induces cell polarization and filopodia (2). This localized actin filament network formation together with substrate adhesion is part of the basic machinery that drives random cell migration. Although the mechanism of random cell migration has been studied in great detail, far less is known about the complex system of regulation that controls directionally persistent migration (3). Directed migration, which can be triggered by extracellular stimuli such as the topography of the extracellular matrix or by a growth factor gradient, is controlled amongst other factors by polarized signalling at the leading edge. The spatial restriction of signalling receptors and pathways at the leading edge stabilizes selected lamellipodia and thereby maintains the orientation and direction of movement (3).

It is now widely accepted that the delivery of plasma membrane components and cell surface receptors to the leading edge via anterograde membrane trafficking pathways is important for lamellipodia extension. Exocytosis of vesicles not only from the endocytic recycling compartment is essential for cell locomotion (4,5) but also the polarized delivery of newly synthesized proteins from the Golgi complex into the leading edge is required for cell polarization and persistent migration (6–8).

In this study, we have focused on the potential role(s) of myosin VI in mammalian cell locomotion. Myosin VI is a unique member of the myosin superfamily, because it moves towards the minus end of actin filaments, in the opposite direction to all other characterized myosins (9). This reverse movement is mediated by a unique 53-amino acid insertion (reverse gear) between the converter region in the motor domain and the neck (lever-arm) region. Myosin VI is ubiquitously expressed in higher eukaryotic cells, where it has been linked to a wide variety of different intracellular functions. In Drosophila, myosin VI is required for various membrane remodelling processes during embryogenesis, suggesting potential roles for myosin VI in cell motility such as in epithelial cell migration during dorsal closure in the embryo (10). Furthermore, in Drosophila ovaries myosin VI is involved in border cell migration, where it interacts and stabilizes β-catenin/E-cadherin adhesion complexes and may also be linked to the formation of membrane protrusions at the leading edge of these moving cells (11).

In mammalian cells, myosin VI plays important roles in endocytic and exocytic membrane trafficking pathways (12–14) as well as in cadherin-based cell–cell contact morphogenesis (15). A number of adaptor molecules have been identified that link myosin VI to different intracellular cargoes associated with specific intracellular functions. For example, myosin VI's function in clathrin-mediated endocytosis at the plasma membrane is mediated by Disabled-2 (Dab2) (16) and the phosphoinositide PIP_2_ Phosphatidylinositol 4,5-bisphosphate (17), whereas GAIP-interacting protein C-terminus (GIPC) (18) and the Ser/Thr transmembrane protein kinase LMTK2 (19) recruit myosin VI to endosomes and the recycling pathway. Recently, GIPC and myosin VI have been linked to α5β1 integrin trafficking (20), suggesting a role for myosin VI in focal adhesion turnover. In addition, myosin VI together with another of its interacting proteins optineurin performs an important role in the secretory pathway for the delivery of cargo from the Golgi complex to the plasma membrane (14,21). Furthermore, in polarized epithelial cells myosin VI and optineurin are required for the sorting of newly synthesized proteins specifically to the basolateral but not to the apical domain (22). In these cells, myosin VI and optineurin can be found on recycling endosomes, which are an important sorting station for cargo *en route* to the basolateral domain (22,23). Sorting of cargo emerging from the *trans* Golgi network and delivery to distinct plasma membrane domains not only occurs in epithelial cells but has also been described for motile cells (24–26). In migrating fibroblasts, proteins with basolateral sorting motifs are transported into the leading edge, whereas in non-motile cells this transport polarity is lost and secretory vesicles are delivered randomly to the entire plasma membrane.

Here, we have investigated the molecular mechanism and the potential roles of myosin VI and its binding partners in cell migration. We conclusively show that myosin VI and its interacting protein optineurin are required for the polarized delivery of epidermal growth factor receptor (EGFR) into the leading edge and that the loss of either myosin VI or optineurin leads to severe defects in protrusion formation. We further show that both proteins are essential for growth factor-stimulated directionally persistent migration, but are not components of the basic machinery that drives random cell migration. Thus, our results indicate that the role of myosin VI in cell migration is directly linked to optineurin and both proteins are required for the delivery of cargo into the leading edge so as to control the spatial distribution of signalling receptors and cell adhesion molecules that are important for the directed movement of cells.

## Results

### Myosin VI is required for lamellipodia and ruffle formation at the leading edge of cells

The leading edge of a motile cell contains membrane protrusions that are formed by actin filament polymerization together with polarized vesicle trafficking (2,27). Myosin VI is enriched in those regions of the plasma membrane in the leading edge that exhibit active lamellipodial protrusions, which characteristically contain membrane ruffles (28).

**Figure 1:**
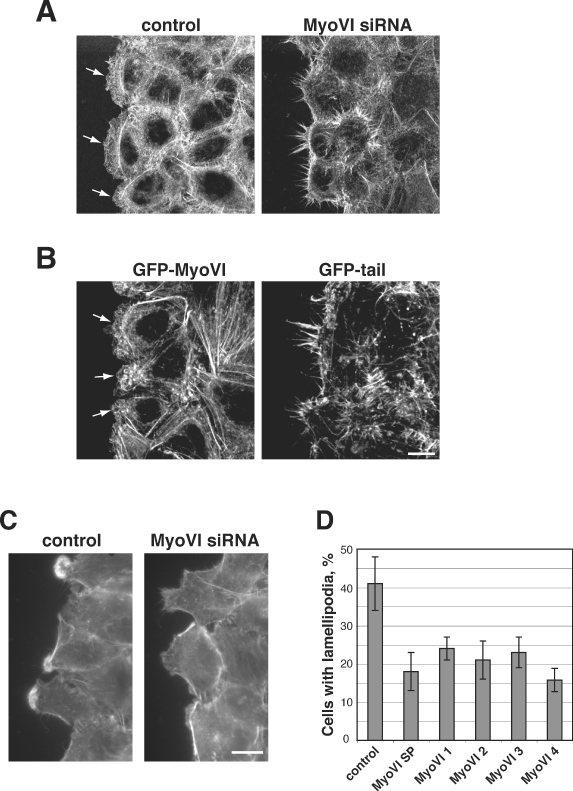
**Inhibition of myosin VI function results in loss of lamellipodia and ruffle formation in HeLa and A549 cells.** A) Control or myosin VI siRNA-treated HeLa cells and (B) HeLa cells stably expressing GFP-myosin VI or GFP-myosin VI tail were grown to confluence, wounded, left to recover for 2 h, then fixed and stained with fluorescently labelled phalloidin. Images represent confocal Z projections. Arrows indicate lamellipodia. Bar, 10 µm. C) A549 cells were transfected with control siRNA or either the smart pool siRNA or four different single siRNA oligos specific to myosin VI. The cells were grown to confluence, wounded, left to recover for 1 h, then fixed and stained with fluorescently labelled phalloidin to label F-actin. Shown is a representative picture of fixed cells at the wound edge. Bar, 10 µm. D) Quantification of the number of control or myosin VI-depleted A549 cells that extend large lamellipodia with extensive ruffles into the open area. Values shown are means ± SD from six independent siRNA experiments (for control and myosin VI smart pool) or means ± range from two independent siRNA experiments (for single oligos).

In this article, we have studied the potential role of myosin VI at the leading edge by investigating the migratory behaviour of cells depleted of myosin VI. We first examined whether myosin VI is required for lamellipodia formation/ruffling during mammalian cell migration. To induce synchronized migration and protrusion formation, a wound was scratched into a confluent layer of HeLa cells, which had been transfected with control or myosin VI siRNA smart pool oligos. Control HeLa cells extended a broad, flat lamellipodium, whereas in myosin VI-depleted cells the lamellipodium is lost and replaced by long finger-like protrusions extending into the open wound area (Figure 1A). Identical results were obtained, when myosin VI functions were inhibited by overexpressing the dominant-negative myosin VI tail domain, i.e. missing the functional motor domain. This approach has been previously shown to inhibit myosin VI-dependent processes in the endocytic pathway and during cytokinesis (12,13,29). HeLa cells overexpressing fully functional full-length green fluorescent protein (GFP)-tagged myosin VI extend broad membrane protrusions at the base of the cell, whereas in cells overexpressing GFP-myosin VI tail there are less lamellipodia and more filopodia emerging into the wound space (Figure 1B).

These results, showing that myosin VI is required for lamellipodia extension and ruffle formation in HeLa cells, were confirmed and quantified in more motile cells, namely A549, the human lung adenocarcinoma epithelial cell line, which extend very prominent lamellipodia that are covered in dorsal ruffles (Figure 1C). These A549 cells were transfected with either control oligonucleotides, smart pool siRNA-targeting myosin VI or with four single oligos specific for myosin VI. A scratch wound was made in a confluent layer of cells and after 1 h the cells were fixed and stained for F-actin. Whereas control A549 cells extend very prominent lamellipodia into the wound (Figure 1C), considerably less of these types of extensions were visible in myosin VI-depleted cells. We counted cells extending large lamellipodia with ruffles into the wound and observed a more than 50% decrease in the ability of myosin VI knockdown (KD) cells to form lamellipodia with ruffles compared to control cells (Figure 1D). Reassuringly, the four single siRNA oligos targeting myosin VI gave a very similar reduction in the number of lamellipodia as compared with cells depleted by the smart pool siRNA (Figure 1D). Thus, in the absence of functional myosin VI, which was depleted either by overexpression of the dominant-negative tail or by siRNA-mediated KD using smart pool siRNA or four single oligos, there is a dramatic reduction in the ability of cells to extend lamellipodia at a scratch wound edge.

### The overall protrusion activity at the plasma membrane is reduced in myosin VI-depleted cells

**Figure 2:**
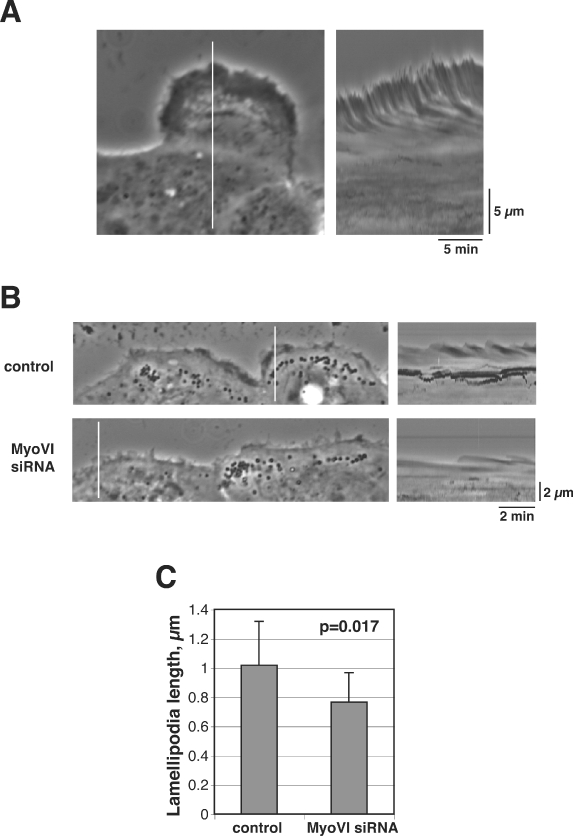
**Depletion of myosin VI affects membrane dynamics at the leading edge.** A) A single frame from a corresponding time-lapse video showing a large lamellipodia at the leading edge of control A549 cell. The image on the right is a kymograph generated from the corresponding video. The vertical white line indicates the area used to generate the kymograph. Ascending contours correspond to membrane protrusion, descending contours correspond to membrane retraction and dark lines indicate membrane ruffling. B) Leading edge dynamics of control and myosin VI siRNA-treated cells. The images on the left represent single frames from corresponding time-lapse series (Videos S1 and S2). Images on the right are kymographs generated from the corresponding videos. C) Quantification of lamellipodia length. Values shown are means ± SD from 30 lamellipodia from at least 10 cells for each condition.

The loss of myosin VI results in a dramatic reduction in the number of A549 cells that are able to extend a large lamellipodium with dorsal ruffles into the space of a scratch wound. The presence or absence of these large lamellipodia was quantified at a single time-point 1 h after applying the scratch wound (Figure 1D). To visualize the overall membrane dynamics, including smaller protrusion activity at the leading edge facing the wound, A549 cells were analysed by time-lapse microscopy. An example of a large lamellipodium (>5 µm on average) that was used to quantify ruffling activity (Figure 1D) is shown in Figure 2A. These large membrane protrusions are present for at least half an hour and show intensive ruffling activity, as can be seen from the corresponding kymograph. We did not observe any significant differences in their overall dynamics such as e.g. their lifetime, which could have explained the reduction in the total number of these large lamellipodia that were detected in fixed cells depleted of myosin VI. However, thorough analysis of the leading edge of the cells facing the wound revealed that depletion of myosin VI leads to a dramatic decrease in the overall protrusion activity of the plasma membrane, which can only be visualized by time-lapse microscopy (Figure 2B and Videos S1 and S2). As can be seen from the kymographs, myosin VI-depleted cells exhibit less plasma membrane fluctuations at the edge of the cell and less membrane ruffles. Comparing the dynamics of smaller lamellipodia protrusions in control and myosin VI KD cells revealed that they had similar lifetimes and protrusion velocities, but the lamellipodia lengths were slightly shorter in myosin VI-depleted cells (Figure 2C). These results indicate that myosin VI is involved in the initiation of lamellipodia protrusion.

### Loss of the myosin VI-interacting protein optineurin reduces lamellipodia formation

**Figure 3:**
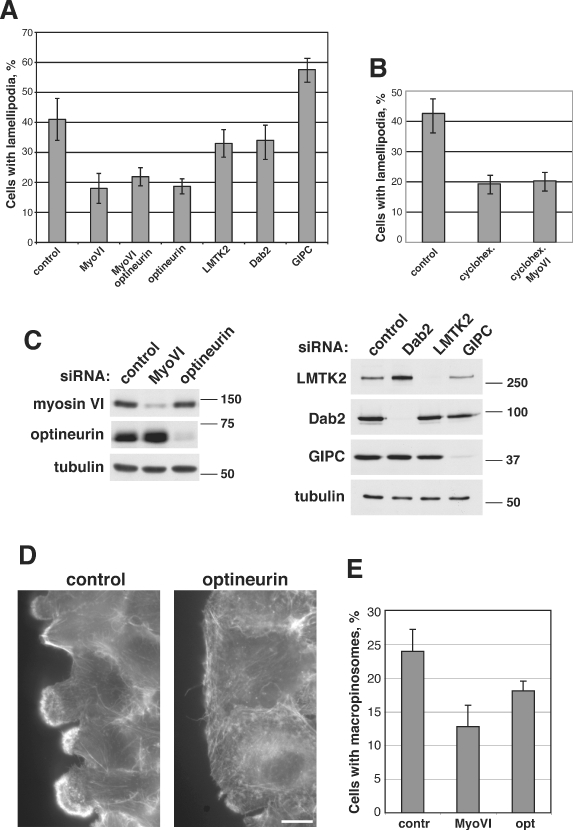
**The myosin VI-interacting protein optineurin is required for lamellipodia and ruffle formation as well as for macropinocytosis in A549 cells.** A549 cells were transfected with control siRNA or siRNA specific to myosin VI, GIPC, optineurin, LMTK2 or Dab2. Two days after the second transfection, the cells were processed for assays. A) A confluent layer of cells was wounded to induce protrusion formation. The graph represents the quantification of the number of cells that extend large lamellipodia with extensive ruffles into the wound. Values shown are means ± SD from six independent siRNA experiments. B) To assess lamellipodia formation in the presence of cycloheximide, control or myosin VI knockdown cells were incubated for 2 h in 50 g/ml of cycloheximide, before the confluent layer of cells was wounded to induce lamellipodia formation in the presence of cycloheximide. C) Western blot of siRNA knockdown experiment with antibodies to optineurin, myosin VI, LMTK2, Dab2 and GIPC. D) A representative picture of fixed control and optineurin knockdown cells at the wound edge. Bar, 10 µm. E) To assess macropinocytosis control, myosin VI or optineurin knockdown cells were incubated with 70-kDa dextran and the number of cells containing dextran-positive structures larger than 0.5 µm in size were counted. Values shown are means ± SD from three independent siRNA experiments.

The intracellular functions of myosin VI are mediated by interacting adaptor proteins, which target myosin VI to different cellular locations and cargoes. Binding partners such as GIPC, Dab2 and LMTK2 link myosin VI to the endocytic pathway, whereas optineurin mediates myosin VI's role in the biosynthetic secretory pathway. To determine which of these binding partners are required in the process of leading edge protrusion and cell migration, we transfected A549 cells with siRNAs specific for GIPC, optineurin, Dab2, LMTK2 or myosin VI and analysed their ability to extend lamellipodia with dorsal ruffles into a scratch wound (Figure 3A). We observed in cells with reduced expression levels of optineurin a 50% reduction in the number of cells extending lamellipodia into the scratch wound. A very similar inhibition of protrusion formation was present in myosin VI KD cells (Figures 1D and 3A). The loss of Dab2 or LMTK2, however, resulted only in a slight drop in lamellipodia formation, whereas in contrast the absence of GIPC did not cause a loss but an increase in the number of cells with lamellipodia at the leading edge (Figure 3A). Even a double KD of Dab2/GIPC, Dab2/LMTK2 or GIPC/LMTK2 did not inhibit protrusion formation indicating that these binding partners involved in endocytic pathways are unlikely to play partially redundant roles in lamellipodia formation (Figure S1A,B). Overall, only the loss of optineurin leads to very similar changes in cell morphology and the absence of lamellipodia extending into the wound as observed in myosin VI KD cells (cf. Figure 1C and Figure 3D). This phenotype is mimicked by the protein synthesis inhibitor cycloheximide, which also causes a 50% inhibition of protrusion formation in control cells. No additional effect was observed in myosin VI KD cells when cycloheximide was added (Figure SB), suggesting that the loss of leading edge protrusions in myosin VI KD cells must be linked to defects in the secretory pathway. Interestingly, the GTPase Rab8, which has been shown to bind to optineurin (30), is not required for protrusion formation (Figure S1A,B).

Membrane ruffles are not only linked to cell motility but are also essential for macropinocytosis, a specialized endocytic pathway that enables the cell to internalize large amounts of fluid from the external environment. Macropinosomes are induced by growth factors and are formed when a circular ruffle closes or folds back onto itself. As macropinosomes are strictly dependent on membrane ruffles, we assessed macropinocytosis in myosin VI and optineurin-depleted cells. The cells were incubated with 70-kDa dextran and the number of cells containing dextran-positive structures larger than 0.5 µm in size was counted. As shown in Figure 3E, the loss of myosin VI or optineurin in A549 cells causes a reduction in the number of cells that have internalized macropinosomes. These results indicate that myosin VI and optineurin are involved in the formation of large sheet-like membrane protrusions and membrane ruffles that are linked to cell motility and macropinocytosis.

### Myosin VI and optineurin are required for polarized delivery of EGFR into the leading edge

**Figure 4:**
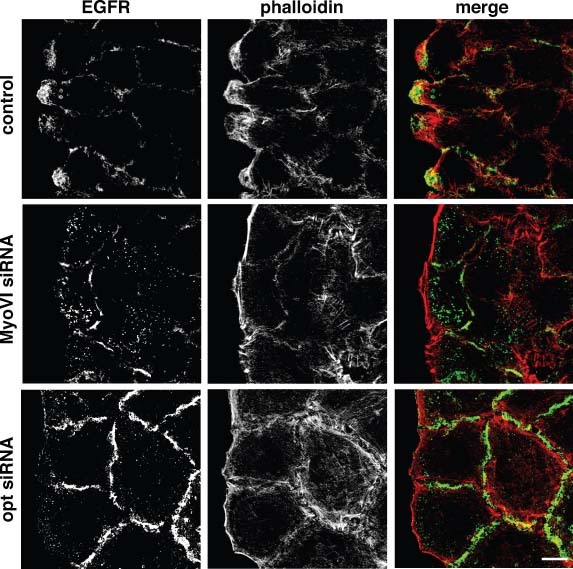
**Myosin VI and optineurin are required for polarized secretion of EGFR into the leading edge.** A549 cells were transfected with control siRNA or siRNA specific to myosin VI or optineurin. The cells were grown to confluence, wounded, left to recover for 1 h, then fixed and processed for immunofluorescence with monoclonal antibody to EGFR and fluorescently labelled phalloidin to label F-actin. Merged colour images are shown in the right-hand panel. The images presented are confocal slices through the base of the cells. Bar, 10 µm.

**Figure 5:**
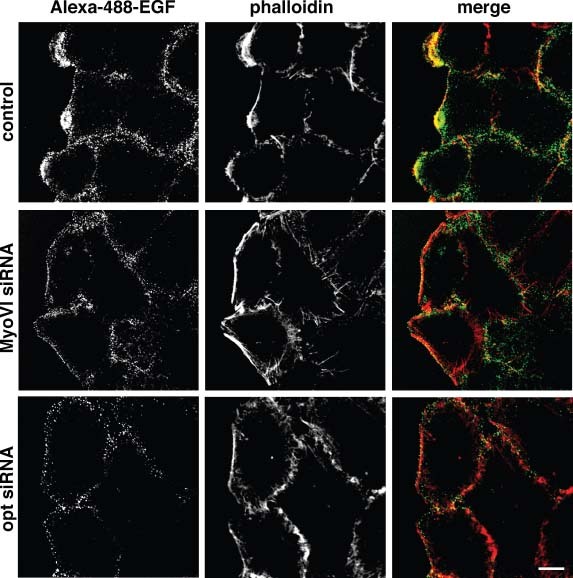
**Loss of myosin VI or optineurin leads to the absence of EGFR from the plasma membrane facing the wound edge.** A549 cells were transfected with control siRNA or siRNA specific to myosin VI or optineurin. A confluent layer of cells was wounded, left to recover for 1 h, incubated with 250 ng/mL Alexa 488-labelled EGF for 3 min, then fixed and stained with fluorescently labelled phalloidin to label F-actin. Merged colour images are shown in the right-hand panel. The images presented are confocal slices through the base of the cells. Bar, 10 µm.

**Figure 6:**
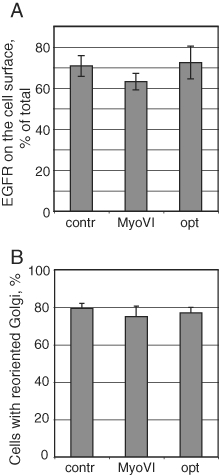
**Depletion of myosin VI or optineurin does not affect steady-state cell surface levels of EGFR or Golgi reorientation.** A) The steady-state surface levels of EGFR in control and myosin VI or optineurin knockdown A549 cells were analysed by FACS as described in *Materials and Methods*. The graph shows the ratio of surface to total antigen for each condition. Values shown are means ± SD from three independent siRNA experiments. B) To assess Golgi reorientation towards the wound, siRNA-treated A549 cells were scratched, fixed after 2 h and processed for immunofluorescence with antibodies to GM130. Cells with Golgi repositioned to face the wound were counted. Values shown are means ± SD from five independent siRNA experiments.

In a migrating cell, secretory vesicles are delivered preferentially towards the leading edge by a process that requires intact microtubules (7) and the positioning of the pericentrosomal Golgi ribbon towards the wound edge (8). In these studies after depolymerization of microtubules or dispersal of Golgi membranes from their pericentrosomal position, normal secretion kinetics were maintained, but the polarized delivery towards the leading edge was lost and defects in directionally persistent migration were observed. As myosin VI and the optineurin-interacting protein Rab8 have been shown to be required for polarized delivery of cargo to the basolateral domain in epithelial cells and towards the synapse in neurons (22,31–33), we tested whether the loss of myosin VI or optineurin affects directed secretion of newly synthesized proteins towards the leading edge. We investigated the localization of EGFR, a receptor tyrosine kinase, in control and KD A549 cells at the edge of a scratch wound. In this cell type, the EGFR is concentrated in membrane protrusions extended into the wound edge, thereby generating a spatial distribution of the receptor supporting localized RTK signalling so as to produce directed cell migration. The concentration of EGFR in lamellipodia can be detected with a monoclonal antibody to the receptor (Figure 4) and also by incubating the cells for short times (<5 min) with fluorescently labelled epidermal growth factor (EGF) (Figure 5). Taken together, both assays indicate that active receptor, which is able to bind EGF, is enriched in membrane protrusion in this cell type (Figures 4 and 5). In the absence of myosin VI or optineurin, however, we observe a dramatic change in the polarized distribution of EGFR at the cell surface lining the wound edge. We specifically detected a loss of EGFR from the plasma membrane facing the wound, but in contrast there was no obvious reduction in the amount of EGFR on the rest of the cell surface such as on lateral membranes that are in contact with neighbouring cells (Figures 4 and 5). This observation was confirmed by fluorescence-activated cell sorter (FACS) analysis, which showed that there was no difference in the amount of EGFR on the total surface of control or KD cells (Figure 6A). Taken together, these results indicate that myosin VI and optineurin are required for the polarized delivery, i.e. the directed secretion of EGFR into the leading edge but not for steady-state exocytosis of these membrane proteins. Interestingly, in myosin VI and optineurin KD cells, but not in control cells, we detected an accumulation of small vesicles in the cytoplasm using a monoclonal antibody to the EGFR (13A9, Genentec) (Figures 4 and S2). This antibody has been reported to bind to both the occupied and empty EGFR (34) and therefore is able to recognize the receptor in the biosynthetic pathway, before it reaches the cell surface. These small EGFR-containing vesicles are not labelled after endocytosis of Alexa 488-EGF, which further confirms the biosynthetic nature of this compartment (Figure S3). In optineurin KD cells, we observed that these EGFR-containing vesicles can cluster into larger aggregates (Figures S2 and S3). To further characterize the nature of these EGFR-containing vesicles in myosin VI KD cells, double labelling experiments were performed with antibodies to marker proteins of the early and late endocytic pathways such as vps26 (retromer complex), cathepsin D and mannose-6-phosphate receptor. None of these marker proteins showed extensive colocalization with this EGFR-containing compartment, supporting our hypothesis that the EGFR-containing vesicles accumulate in the exocytic and not in the endocytic pathway (Figure S4). Interestingly, also the delivery of β1-integrin towards the front of the cell is affected in myosin VI or optineurin KD cells (Figure S5).

As polarized delivery into the leading edge is also dependent on Golgi positioning, we investigated whether Golgi reorientation towards the wound is affected in myosin VI or optineurin KD cells. In A549 cells, 1.5–2 h after wounding 80% of the Golgi complexes are repositioned to face the wound edge and reduction in myosin VI or optineurin expression does not result in a significant defect in Golgi reorientation in the KD cells (Figure 6B).

### Loss of myosin VI or optineurin does not affect random cell migration

**Figure 7:**
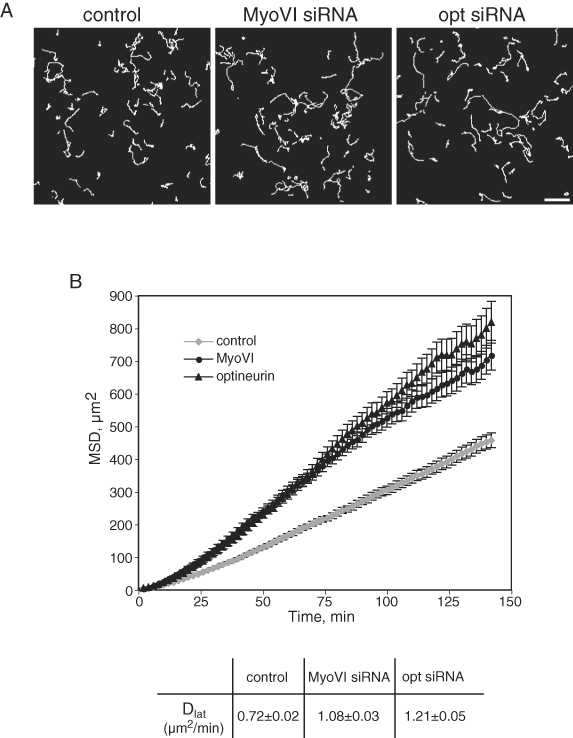
**Depletion of myosin VI or optineurin leads to changes in random cell migration.** To analyse the random migration behaviour of A549 cells, we used time-lapse microscopy to capture the movements of individual cells on fibronectin-coated glass. A) Randomly selected fields of migration tracks of control, myosin VI or optineurin siRNA-treated cells tracked for 5 h. Bar, 100 µm. B) MSD of cells calculated from *XY* coordinates over time. The migration tracks of 3434 control, 2577 myosin VI and 1950 optineurin KD cells were analysed combined from four (for myosin VI) or three (for optineurin) independent siRNA experiments. Error bars represent SD. The table represents the diffusion coefficients (*D*_lat_± SD) calculated from MSD curve fitting.

To determine whether myosin VI or optineurin depletion has any effect on random single cell migration in an isotropic environment, A549 cells were plated onto fibronectin-coated glass chambers, time-lapse videos collected and the movements of individual cells were followed using tracking software. The tracks of 3434 control cells, 2577 myosin VI siRNA-treated cells and 1950 optineurin siRNA-treated cells were analysed. Still images of time-lapse microscopy videos of control or siRNA-treated A549 cells with migration tracks superimposed onto the original image sequences are shown in Figure 7A. Measuring the individual trajectories of the cell centroids and subsequent analysis of the cell movements showed that myosin VI or optineurin depletion results in an increase in the average diffusion coefficient from 0.72 ± 0.02 (control cells) to 1.08 ± 0.03 µm^2^/min (myosin VI KD cells) and 1.21 ± 0.05 µm^2^/min (optineurin KD cells), which corresponds to the steeper mean-squared displacement (MSD) curves shown in Figure 6. Both myosin VI (0.72 ± 0.04 µm/min) and optineurin KD cells (0.48 ± 0.01 µm/min) move faster with higher velocity as compared with control cells (0.37 ± 0.01 µm/min). These results indicate that the loss of myosin VI or optineurin does not affect the basic ability of cells to perform random movements. On the contrary, myosin VI and optineurin KD cells show an increase in non-directional random motility (Figure 7).

### Myosin VI and optineurin are requiredfor chemotactic migration

**Figure 8:**
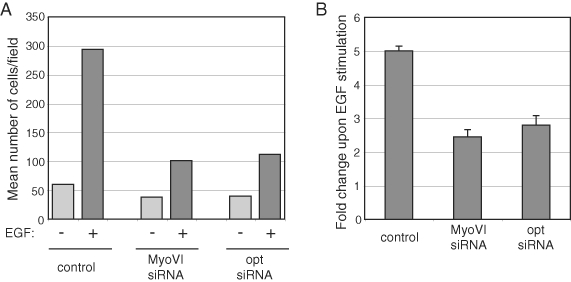
**Myosin VI and optineurin are required for directed migration towards an EGF gradient.** SiRNA-treated A549 cells were starved and plated into the upper chambers of Transwells in serum-free medium and the same medium with or without 20 ng/mL EGF was added to the lower well. After 5 h the cells were fixed, stained and the cells on the under surface were counted. A) A representative experiment showing the number of control and myosin VI or optineurin siRNA-treated cells migrated under basal (light grey bars) and EGF-stimulated (dark grey bars) conditions. B) Chemotactic response to EGF. The graphs represent the fold increase in migration, calculated as a ratio of the number of cells migrated to the under surface of the membrane upon EGF stimulation to the number of cells migrated under basal conditions. Values shown are means ± SD from three independent siRNA experiments.

The directed movement of cells is dependent on spatially restricted receptor tyrosine kinase signalling. As the polarity of EGFR localization is lost in myosin VI and optineurin KD cells, we tested the ability of myosin VI or optineurin KD cells to migrate towards a chemotactic stimulus using a Transwell migration assay. SiRNA-treated A549 cells were plated in the upper chambers of Transwells and the lower chambers were filled with medium with or without 20 ng/mL of EGF. In the absence of EGF, the number of control cells that had migrated after 5 h to the underside of the filter was similar to the number of myosin VI or optineurin KD cells. However, in the presence of EGF in the lower chamber, control cells responded with a fivefold increase in the number of cells migrating towards a chemotactic gradient, whereas in myosin VI or optineurin-depleted cells only a 2.5-fold or 2.8-fold increase in migration was observed (Figure 8A,B). These results indicate that both myosin VI and optineurin are required to sustain directed migration towards a chemotactic stimulus, and the mechanism involved is the polarized trafficking of membrane components into the leading edge of the cell.

## Discussion

We have shown that myosin VI and its interacting protein optineurin are essential for directed cell migration in mammalian cells. The loss of either optineurin or myosin VI inhibits the delivery of EGFR to the leading edge at the front of the cell and causes a dramatic defect in lamellipodia formation. Surprisingly, this does affect random migration of myosin VI or optineurin KD cells; however, both proteins are crucial for cells to perform directed migration towards a chemotactic gradient. As optineurin has been shown to mediate the function of myosin VI in post-Golgi membrane transport, our results indicate that the role of myosin VI in cell motility is linked to anterograde membrane trafficking pathways.

Numerous lines of evidence now suggest that in a motile polarized cell plasma membrane proteins are delivered into the leading edge in an anterograde direction from the Golgi complex and/or the endocytic recycling pathway. In this process, directed secretion towards the wound edge is dependent on Golgi positioning; however, very little is known about the other constituents of the machinery responsible for polarized delivery into the leading edge. Interestingly, in neither myosin VI or optineurin KD cells was a defect in Golgi reorientation towards the wound observed, indicating that myosin VI and optineurin are not involved in this process, but are most likely involved in polarized post-Golgi transport or sorting steps. Furthermore, as cdc42 is the key regulator of cell polarity and Golgi reorientation, our results suggest that myosin VI and optineurin are not major players in this cdc42-dependent signalling pathway. This hypothesis is supported by the finding that depletion of functional myosin VI and optineurin leads to a reduction of Rac-dependent lamellipodia but to an increase in finger-like protrusions known as filopodia, which in most cell types are formed after cdc42 activation. This replacement of broad lamellipodia and ruffles with finger-like filopodia can be observed both in myosin VI-depleted cells in scratch wound assays of cell monolayers (Figure 1) and also during single cell spreading in GFP-myosin VI tail-expressing cells. Interestingly, these dramatic morphological changes do not result in changes in the speed of spreading of myosin VI-depleted cells (data not shown).

In epithelial cells, distinct pathways for the selective sorting of cargo leaving the *trans* Golgi network to either the apical or basolateral cell surface have been described. In polarized MDCK cells, we have shown a role for myosin VI and optineurin in the selective sorting of cargo such as vesicular stomatitis virus glycoprotein (VSV-G) to the basolateral domain (22). In a motile fibroblast, VSV-G is targeted to the leading edge (35), suggesting that similar pathways and machinery used for delivery of proteins to the basolateral domain are also required for targeting of proteins to the leading edge in motile cells. Interestingly in neurons, trafficking in the biosynthetic pathway is also targeted to distinct subdomains of the plasma membrane, e.g. to the dendritic surface of the cell body, which is similar to the basolateral domain and to the axonal membrane, which can be regarded as analogous to the apical domain of MDCK cells (24). Thus, in hypocampal neurons, exocytic trafficking of AMPA (α-amino-3-hydroxy-5-methyl-4-isoxazolepropionic acid) receptor to the postsynaptic membrane at the somatodendritic cell surface, the basolateral analogue, has been shown to require myosin VI (33). These observations give further support to our observations that a complex of myosin VI and optineurin drives the local delivery of EGFR into the leading edge of a motile cell at the wound edge.

Previously, myosin VI and optineurin have been linked to constitutive exocytosis of reporter molecules such as secreted alkaline phosphatase (SEAP) and VSV-G; depletion of either myosin VI or optineurin resulted in a kinetic delay in delivery of the reporters to the cell surface (14,21). In this study, we show that the steady-state surface levels of endogenous transmembrane proteins, such as the EGFR, are not affected in myosin VI or optineurin KD cells. The absence of myosin VI or optineurin, however, dramatically impairs the selective delivery of these membrane proteins into the leading edge. These results indicate that myosin VI and optineurin are required for the spatial delivery of membrane proteins in the anterograde trafficking pathway, but are not essential for steady-state secretion of every cargo to the plasma membrane. Transmembrane proteins are either delivered directly from the Golgi complex to the plasma membrane or via an intermediate recycling endosome. In polarized epithelial cells, myosin VI and optineurin are present on a specialized recycling compartment, a sorting station for proteins delivered to the basolateral domain (22). In epithelial cells, cargo *en route* to the basolateral domain can pass through a possibly specialized type of recycling endosome, and also in macrophages this type of organelle is important for polarized secretion of cytokines (36). This proposed role of myosin VI and optineurin in anterograde trafficking pathways, which include the route from the recycling compartment to the plasma membrane, is supported by our finding that polarized delivery of β1-integrin towards the cell front is also affected in myosin VI or optineurin A549 KD cells (Figure S5). Future work will confirm whether the myosin VI-dependent delivery of the EGFR to the leading edge involves a sorting step in a recycling compartment, whereas transport to the rest of the plasma membrane proceeds directly from the *trans* Golgi network and therefore may not be dependent on myosin VI.

Interestingly, the absence of myosin VI or optineurin, which leads to a dramatic reduction in lamellipodia formation at the leading edge, does not lead to defects in random cell migration. In contrast, myosin VI or optineurin-depleted cells show an increase in motility, which is reflected in an increase in MSD as well as the velocity over time. Although it seems surprising that myosin VI and optineurin KD cells with less lamellipodia show increased random migration, this is not a new observation. In epithelial PtK1 cells, non-muscle tropomyosin inhibition causes a loss of lamellipodia, but a dramatic increase in random migration velocity (37), supporting our observation that indeed cells can migrate without a lamellipodium.

Although myosin VI or optineurin KD cells do not lack the ability to move, they are impaired in performing directed migration towards a stimulus gradient. This type of movement requires a signal perceived and amplified in a restricted area at the front of the cell. Although a polarized concentration of signalling receptors at the leading edge is not required in all cell types for chemotaxis (38), we clearly observed an accumulation of EGFR in the plasma membrane of protrusions at the front of A549 cells close to the coverslip using confocal microscopy. Therefore, we believe that the polarized localization of EGFR at the leading edge of A549 cells enhances localized signalling at the front, which is important for directed migration (39). Polarized secretion has also been shown in migrating fibroblasts, where secretory vesicles are preferentially delivered and inserted at the leading edge (7).

Our results are consistent with previous work in Drosophila that implicated myosin VI in epithelial cell migration required for dorsal closure during embryogenesis (10) and in border cell migration in oocytes (11). In these border cells, inhibition of myosin VI also leads to a reduction in protrusion formation at the leading edge. From Drosophila oocytes, myosin VI can be isolated in a complex with Drosophila E-cadherin and it was therefore proposed that myosin VI promoted protrusion formation by moving towards the minus end of the polymerizing actin filaments while binding to a stationary E-cadherin complex and thereby pushing the actin filaments into the leading edge. At present, we cannot exclude this role for myosin VI in protrusion formation in mammalian cells; however, we did not observe any association of myosin VI with focal adhesion complexes at the leading edge in A549 cells or any other defects in focal adhesion formation or cell spreading in myosin VI-depleted cells. Our results, however, are supported by data showing that although vinculin is a downstream effector of myosin VI at E-cadherin-dependent cell–cell contact sites, no changes or defects at integrin-based cell-matrix adhesion sites were observed upon myosin VI depletion (15). In contrast, a recent study indicates that in endothelial cells myosin VI and its interacting protein GIPC are important for α5β1 integrin endocytosis and that the absence of either protein causes defects in cell adhesion (20). These different results suggest that there are probably cell-specific variations in the involvement of myosin VI in cell adhesion formation.

In addition, we cannot exclude a role for myosin VI in actin filament reorganization at the leading edge, although so far no interacting protein directly linking myosin VI to the machinery regulating actin filament dynamics has been identified in mammalian cells. However, we have previously shown the recruitment of myosin VI into the leading edge after EGF stimulation (28). Furthermore, in Drosophila myosin VI has been shown to stabilize actin filaments required for spermatid individualization (40). Therefore, future work is required to show whether myosin VI coordinates membrane delivery and actin organization at the leading edge.

In summary, we have clearly shown that myosin VI together with the adaptor protein optineurin is required for the delivery of specific cargo such as the EGFR in the anterograde pathway towards the front of a migrating cell. This delivery is essential for protrusion formation at the leading edge of the cell and for the generation of directionally persistent migration.

## Materials and Methods

### Plasmid constructs and antibodies

Constructs coding for GFP-tagged full-length human myosin VI and the tail domain were described previously (19,29). To generate constructs for stable expression, GFP-MyoVI and GFP-tail fusion cassettes were subcloned from pEGFPC3 into the pIRESneo2 vector (Clontech).

The following commercial antibodies were used: rabbit polyclonal antibodies to GFP (Molecular Probes), mouse monoclonal antibodies to GM130 (BD Transduction Laboratories) and β1 integrin (P5D2; Santa Cruz), α-tubulin (Sigma); 13A9 monoclonal to EGFR was kindly provided by Genentec. Antibodies to myosin VI and optineurin were previously described (21,28).

### Cell culture, transfection and siRNA

HeLa cells were cultured in RPMI-1640 and A549 in DMEM, containing 10% FCS, 2 mml-glutamine, 100 U/mL penicillin and 100 µg/mL streptomycin, and transfected using FuGENE (Roche Diagnostics), according to the manufacturer's instruction. To generate GFP-MyoVI and GFP-tail stably expressing cell lines, HeLa cells were transfected with corresponding constructs in pIRESneo2 and selected with complete medium containing 500 µg/mL G418 (Gibco). All stable clones were combined for each construct and the cells were FACS sorted to enrich for highly expressing cells.

All siRNA oligos were obtained from Dharmacon. ON-TARGETplus smart pool as well as single ON-TARGETplus oligos (19) were used to KD myosin VI. ON-TARGETplus smart pool and a single duplex (21) were used for depletion of optineurin. Smart pool and single oligos yielded similar levels of KD and the same phenotypes. ON-TARGETplus smart pool were used for KD of GIPC and Dab2, single duplex 2068 (19) was used for KD of LMTK2, a non-targeting oligo was used as a control. For efficient KD, HeLa and A549 cells were transfected twice with siRNA on day 1 and 3 using Oligofectamine (Invitrogen). On day 5, cells were processed for corresponding assays and the efficiency of protein depletion was assessed by western blotting.

### Immunofluorescence

HeLa cells growing on coverslips were transfected with corresponding plasmids and 24 h later were fixed with 4% paraformaldehyde (PFA), permeabilized with 0.1% Triton-X-100, blocked with 1% BSA in PBS and processed for indirect immunofluorescence using primary antibodies (specified in the figure legends) and secondary antibodies coupled with Alexa 488 or Alexa 568 and Alexa 568-labelled phalloidin (Molecular Probes).

For scratch wound assays, siRNA-treated HeLa or A549 cells, or HeLa cells stably expressing GFP-myosin VI or GFP-tail, were plated on coverslips at high density and 24 h later scratched with a pipette tip. The cells were incubated in growth medium and fixed after 2 h (HeLa) or after 1 h (A549) and processed for immunofluorescence as described earlier.

For macropinocytosis assay, siRNA-treated A549 cells were plated on coverslips at low density and next day were starved for 6 h and loaded with 0.5 mg/mL of 70-kDa tetramethylrhodamine-coupled lysine-fixable dextran (Molecular Probes) for 30 min in the presence of 20 ng/mL EGF, then fixed and mounted for microscopy. The cells were visualised with a Zeiss Axioplan epifluorescence microscope equipped with 63×/1.4 numerical aperture (NA) objective and a Hamamatsu ORCA-R2 charge-coupled device (CCD) camera driven by Simple PCI software. Alternatively, Zeiss LSM-510 confocal microscope (Carl Zeiss MicroImaging Inc.) was used. Images were processed in Adobe Photoshop and assembled in Adobe Illustrator.

### Immunoblotting

Cells were lysed in SDS–PAGE sample buffer and the lysates were analysed by SDS–PAGE followed by immunoblotting using standard protocol. Blots were developed using ECL detection reagent (GE Healthcare).

### Live-cell imaging and analysis of membrane dynamics

A549 cells growing on coverslips were wounded with a pipette tip, washed and left to recover at 37°C in CO_2_-independent medium supplemented with 10% FCS. Time-lapse videos were acquired between 1 and 2 h after wounding using a Zeiss Axiovert 200M microscope equipped with a heated stage, 60× oil phase-contrast objective and a Photometrics Cool Snap CCD camera (Roper Scientific) driven by OpenLab software (Improvision). Images were captured with 2- or 5-second intervals. The dynamics of lamellipodial extensions was analysed using Volocity software (Improvision). Kymographs were created from time-lapse sequences and the following parameters were calculated: maximum height of individual peaks (corresponding to lamellipodia length), maximum width of individual peaks (corresponding to lamellipodia lifetime) and the slope of the ascending contours (corresponding to protrusion velocity).

### Flow cytometry

Steady-state surface and total EGFR and β1 integrin levels were measured by flow cytometry. A549 cells were detached using non-enzymatic cell dissociation solution (Invitrogen), pelleted by centrifugation, washed with medium, cooled down on ice and incubated with antibodies to EGFR or β1 integrin on ice for 30 min. Unbound antibodies were washed off with ice-cold PBS, the cells were then fixed in 4% PFA for 20 min and permeabilized with 0.1% Triton-X-100 in PBS for 5 min at room temperature. At this point, each sample was split into two, one half (representing surface amount of the antigen) was incubated with Alexa 488-conjugated goat anti-mouse secondary antibody for 30 min at room temperature and the second half (representing total amount of the antigen) was incubated again with the corresponding primary and then with secondary antibody. Cell-associated Alexa 488 fluorescence was determined using BD FACSCalibur flow cytometer (BD Biosciences). Cells labelled with secondary antibody only were used as negative controls in each experiment. All experiments were performed in duplicates and repeated three times. Mean fluorescence intensity was calculated using FlowJo software.

### Migration assays

To assess random migration, A549 cells were labelled with 5 µm of Cell Tracker CMRA (Invitrogen) for 30 min according to the manufacturer's instructions. Labelled cells were dissociated using accutase (PAA Laboratories) and resuspended in DMEM supplemented with 10% FCS, glutamine and 10 mm HEPES. The cells were plated onto a fibronectin-coated coverglass system (chambered 1.0 borosilicate; Lab-Tek) at a density of 20 000 cells/cm^2^ and left in the incubator for 2 h before time-lapse analysis. Cells were visualized using an RT live-imaging workstation (Deltavision; Applied Precision, LLC) and an Olympus IX-70 microscope equipped with a CCD camera (Micromax 1300 YHS; Roper Scientific) in a heated environmental chamber (37°C, 5% CO_2_). Images of cells were taken with 4×/0.13 NA objective and Rhodamine-Texas Red filter set (Chroma Technology Corp.) with 2-min intervals for 4 h using Softworx acquisition software (Applied Precision, LLC). Myosin VI or optineurin siRNA-treated cells were imaged simultaneously with controls; 3434 control, 2577 MyoVI and 1950 optineurin siRNA cell tracks were analysed combined from four (for myosin VI) or three (for optineurin) independent siRNA KD experiments. Measurement of *XY* coordinates of cells centroids and subsequent analysis of cell movement were performed using single particle tracking algorithm in Gmimpro software (41), http://www.nimr.mrc.ac.uk/gmimpro/. MSD of cells was calculated using overlapping intervals. The cell speed was calculated by fitting the MSD data into the random walk equation.

For Transwell migration assays, 24-well Falcon chambers with 8-µm pore membrane (Beckton Dickinson Labware) were used. Both sides of the membrane were coated with 10 µg/mL collagen I (Sigma). A549 cells were starved overnight and seeded to the upper chambers at a density of 1 × 10^5^/well in DMEM containing 0.1% BSA. The same medium with or without 20 ng/mL EGF was added to the lower well and the cells were allowed to migrate for 5 h at 37°C. At the end of the assay, the inserts were fixed and stained using Hema Gurr rapid staining set (BDH), cells from the upper surface were removed with a cotton swab and the cells migrated to the under surface were counted under low magnification. At least 10 independent fields were counted for each membrane, all experiments were performed in duplicates and repeated three times.

### Statistical analysis

Statistical evaluations were performed using two-sample Kolmogorov–Smirnov tests calculated in statistical package R (http://www.r-project.org).

## Supporting Information

**Figure S1: Lamellipodia formation in A549 cells.** A) To assess lamellipodia formation, A549 cells were transfected with control siRNA, siRNA specific for Rab8 or for double knockdowns with siRNA specific for myosin VI and optineurin, for Dab2 and GIPC, for LMTK2 and Dab2 or for LMTK2 and GIPC. The wound healing assay was performed as described in Figure 3. The graph shows a representative experiment presenting the percentage of cells that extend large lamellipodia into the wound. B) Western blot of siRNA knockdown experiment using antibodies to LMTK2, myosin VI, Dab2, optineurin, GIPC and Rab8.

**Figure S2: Loss of myosin VI or optineurin causes accumulation of EGFR-containing vesicles.** A549 cells were transfected with control siRNA or siRNA specific to myosin VI or optineurin. A confluent layer of cells was wounded, fixed after 1 h when maximal protrusion formation was observed and stained with a monoclonal antibody to EGFR and fluorescently labelled phalloidin to label F-actin. Merged colour widefield images are shown in the right-hand panel. Bar, 10 µm.

**Figure S3: EGFR-positive vesicles are only partially labelled after endocytosis of fluorescently labelled EGF.** A549 cells were transfected with control siRNA or siRNA specific to myosin VI or optineurin and incubated with 250 ng/mL Alexa 488-labelled EGF for 10 min before fixing and staining with the monoclonal antibody to EGFR. The images presented are widefield. The merged colour images are shown in the right-hand panel. Bar, 10 µm.

**Figure S4: EGFR-containing vesicles do not colocalize with marker proteins of the endocytic pathway.** A549 cells were transfected with control siRNA or siRNA specific to myosin VI. After fixation, the cells were double labelled with the monoclonal antibody to the EGFR and different marker proteins of the endocytic pathway: cathepsin D to label lysosomes, mannose-6-phosphate receptor (M6PR) to label late endosomes and vps26, a component of the mammalian retromer complex, which is present on early and late endosomes. Bar, 10 µm.

**Figure S5: Myosin VI and optineurin are required for targeting of β1-integrin to the plasma membrane facing the wound.** A) A549 cells were transfected with control siRNA or siRNA specific to myosin VI or optineurin. The cells were grown to confluence, wounded, left to recover for 1 h, then fixed and processed for immunofluorescence with monoclonal antibody to β1-integrin and fluorescently labelled phalloidin to label F-actin. Merged colour images are shown in the right-hand panel. The images presented are confocal slices through the base of the cells. B) The steady-state surface levels of β1-integrin in control and myosin VI or optineurin knockdown A549 cells were analysed by FACS as described in *Materials and Methods*. The graph shows the ratio of surface to total antigen for each condition. Values shown are means ± SD from three independent siRNA experiments. Bar, 10 µm.

**Video S1:** Time-lapse phase-contrast video of control siRNA-treated A549 cell at the wound edge. Images were taken every 2 seconds for 8.5 min.

**Video S2:** Time-lapse phase-contrast video of myosin VI-depleted A549 cell at the wound edge. Images were taken every 2 seconds for 8.5 min.
