# Supplementary figures and images for: Myosin VI and Optineurin Are Required for Polarized EGFR Delivery and Directed Migration
Source: Traffic. 2010 Aug 3;11(10):1290–303. doi: 10.1111/j.1600-0854.2010.01101.x (PMC3039242; doi:10.1111/j.1600-0854.2010.01101.x)

**A**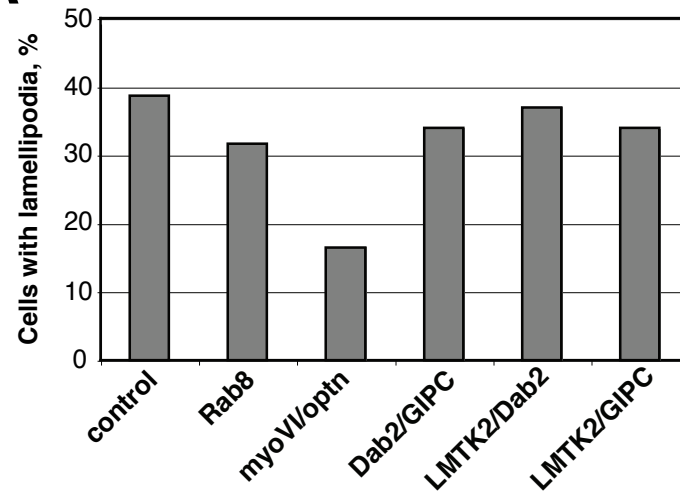**B**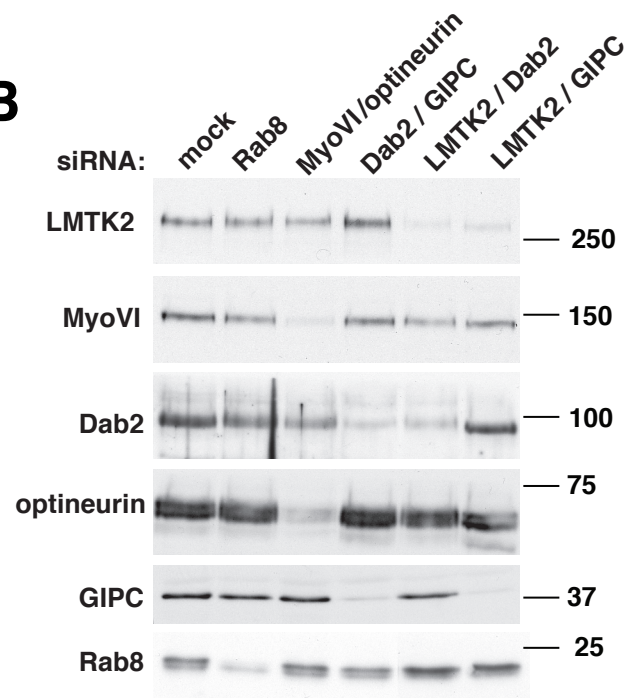

Supplementary figure 1, Chibalina et al

Supplement: Supplementary file 1 [file tra0011-1290-SD1.pdf]

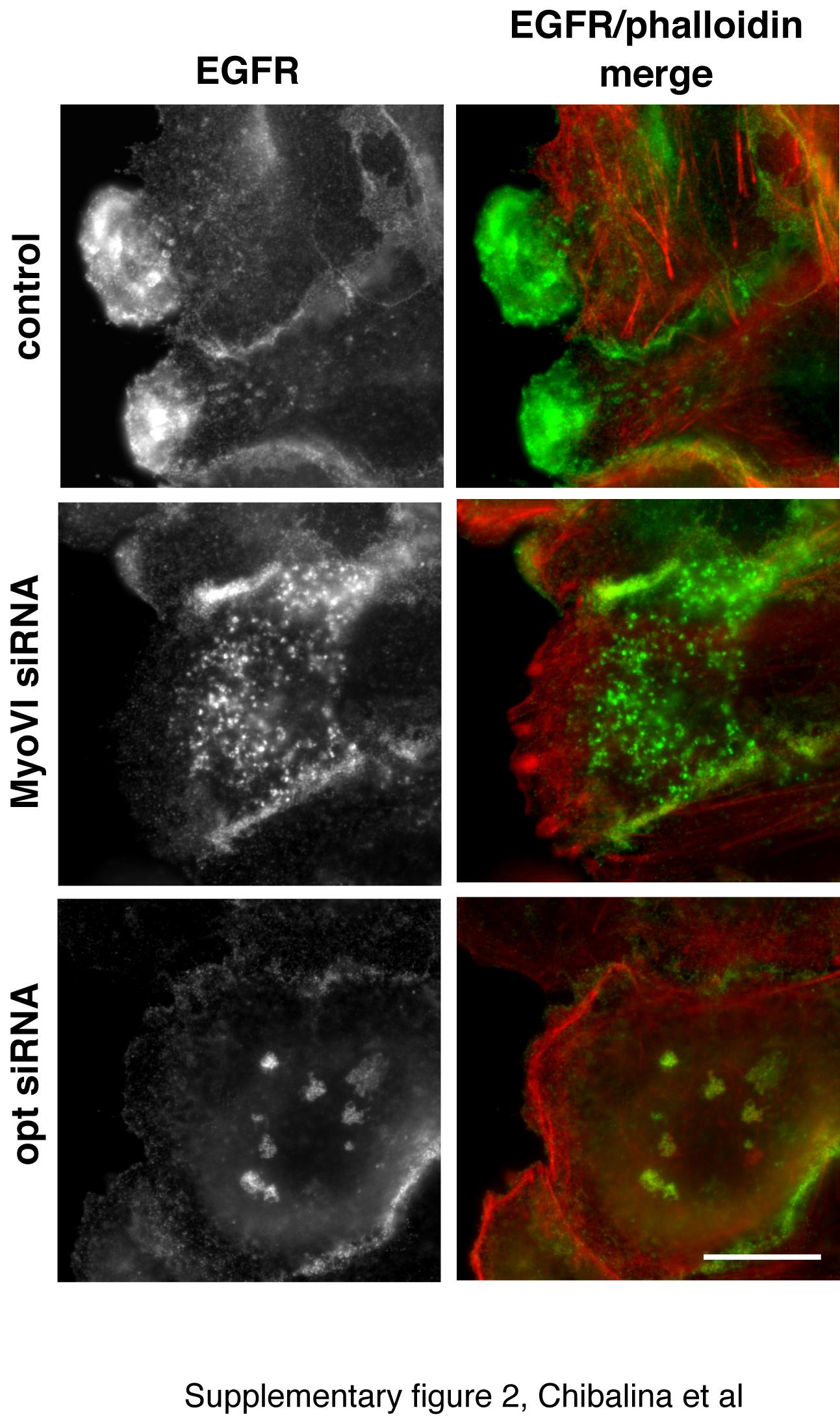

Supplement: Supplementary file 2 [file tra0011-1290-SD2.tif]

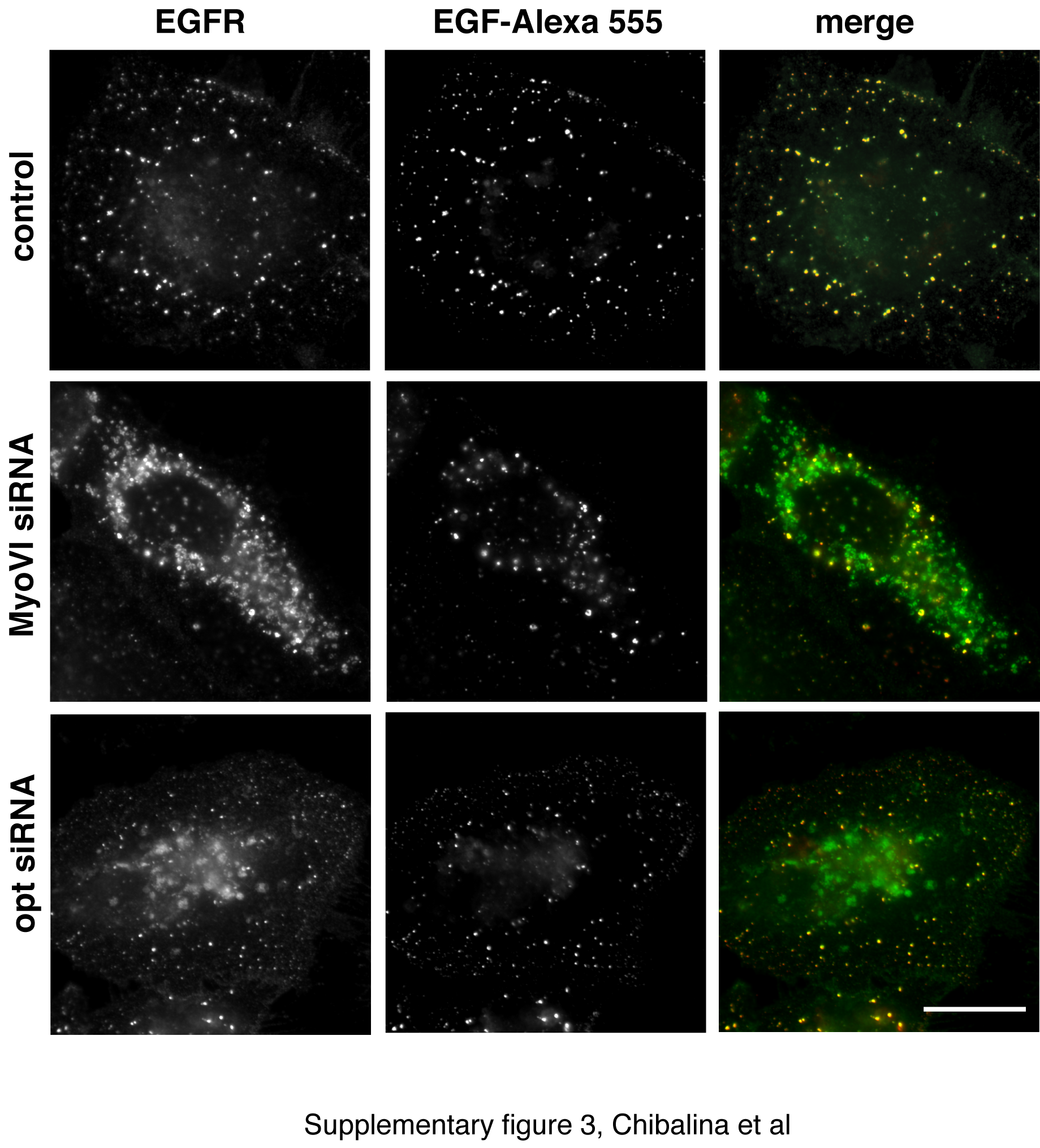

Supplement: Supplementary file 3 [file tra0011-1290-SD3.tif]

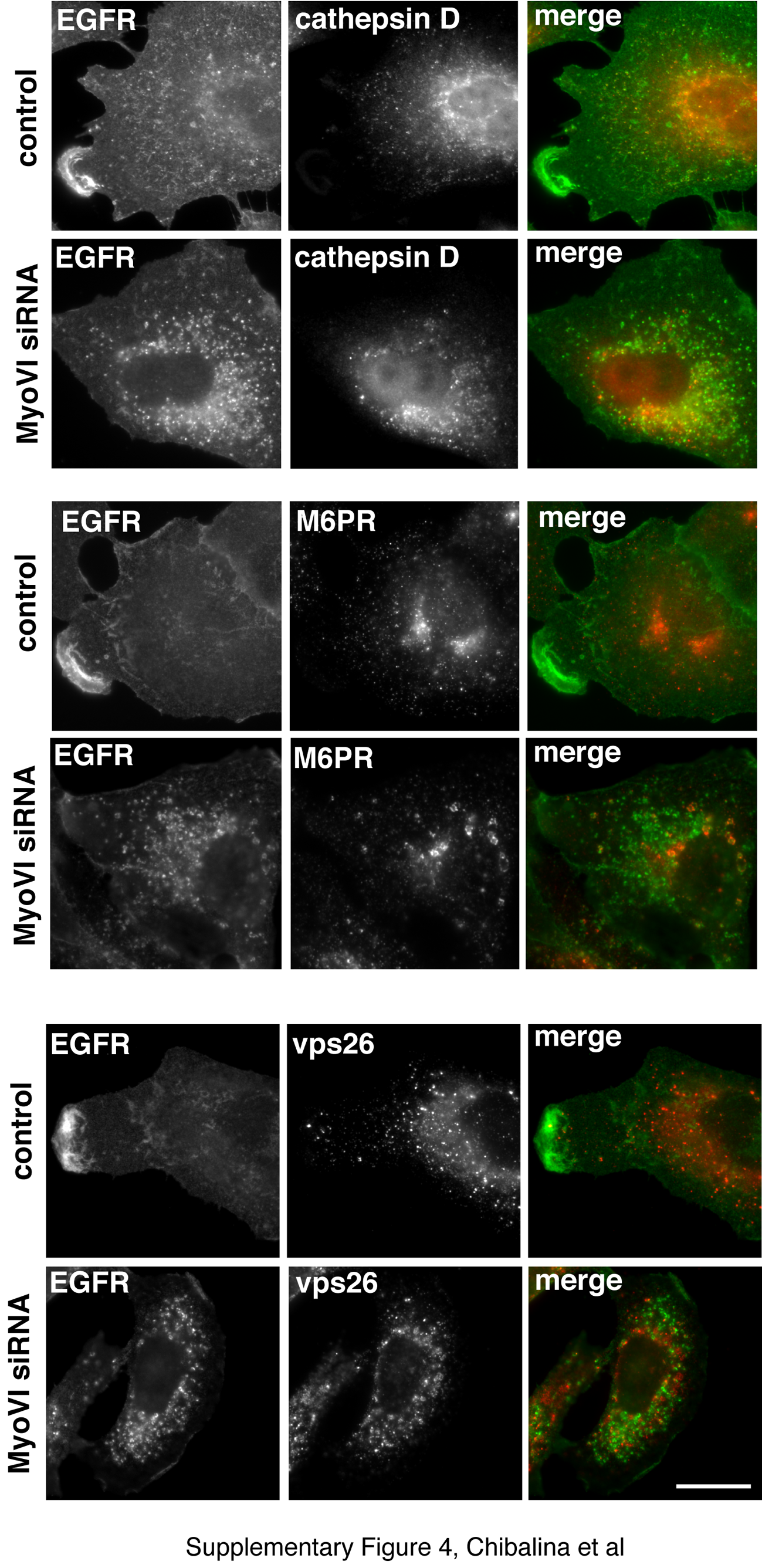

Supplement: Supplementary file 4 [file tra0011-1290-SD4.tif]

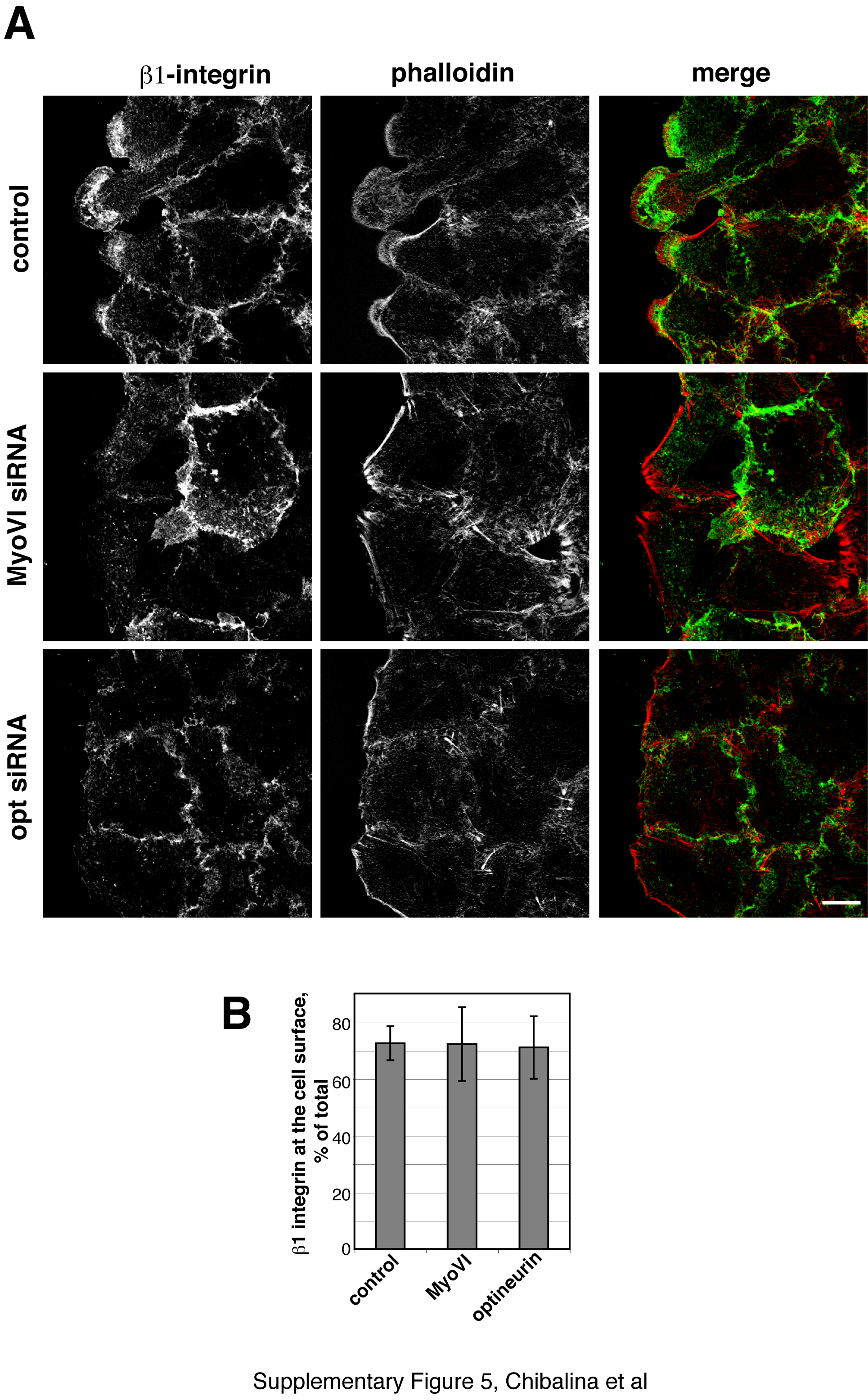

Supplement: Supplementary file 5 [file tra0011-1290-SD5.tif]
